# Cardio- and Neurotoxicity of Selected Anti-COVID-19 Drugs

**DOI:** 10.3390/ph15060765

**Published:** 2022-06-20

**Authors:** Martin W. Nicholson, Ching-Ying Huang, Jyun-Yuan Wang, Chien-Yu Ting, Yu-Che Cheng, Darien Z. H. Chan, Yi-Chan Lee, Ching-Chuan Hsu, Yu-Hung Hsu, Cindy M. C. Chang, Marvin L. Hsieh, Yuan-Yuan Cheng, Yi-Ling Lin, Chien-Hsiun Chen, Ying-Ta Wu, Timothy A. Hacker, Joseph C. Wu, Timothy J. Kamp, Patrick C. H. Hsieh

**Affiliations:** 1 Institute of Biomedical Sciences, Academia Sinica, Taipei 115, Taiwan; mawnicho@gmail.com (M.W.N.); jenniferhuang0820@gmail.com (C.-Y.H.); chienyu@ibms.sinica.edu.tw (C.-Y.T.); criss1125@ibms.sinica.edu.tw (Y.-C.C.); darien.czh@ibms.sinica.edu.tw (D.Z.H.C.); paul84114@gmail.com (Y.-C.L.); gingerhsu0904@gmail.com (C.-C.H.); hsuyuhung@yahoo.com (Y.-H.H.); mlhsieh@wisc.edu (M.L.H.); natascha_yuan@yahoo.com (Y.-Y.C.); yll@ibms.sinica.edu.tw (Y.-L.L.); chchen@ibms.sinica.edu.tw (C.-H.C.); 2 Genomics Research Center, Academia Sinica, Taipei 115, Taiwan; jyunyuanwang@gate.sinica.edu.tw (J.-Y.W.); ywu@gate.sinica.edu.tw (Y.-T.W.); 3 Cardiovascular Physiology Core Facility, Department of Medicine, University of Wisconsin-Madison, Madison, WI 53705, USA; cchang9336@gmail.com (C.M.C.C.); th2@medicine.wisc.edu (T.A.H.); 4 Stanford Cardiovascular Institute, Stanford University School of Medicine, Stanford, CA 94305, USA; joewu@stanford.edu; 5 Department of Medicine and Stem Cell and Regenerative Medicine Center, University of Wisconsin-Madison, Madison, WI 53705, USA; tjk@medicine.wisc.edu; 6 Institute of Clinical Medicine, National Taiwan University, Taipei 106, Taiwan

**Keywords:** stem cell research, cardiomyocyte, neuron, drug screening, human leukocyte antigen, human-induced pluripotent stem cell, toxicity, COVID-19

## Abstract

Since December 2019, the novel coronavirus disease 2019 (COVID-19), caused by severe acute respiratory syndrome coronavirus 2 (SARS-CoV-2), has infected ~435 million people and caused ~6 million related deaths as of March 2022. To combat COVID-19, there have been many attempts to repurpose FDA-approved drugs or revive old drugs. However, many of the current treatment options have been known to cause adverse drug reactions. We employed a population-based drug screening platform using 13 human leukocyte antigen (HLA) homozygous human induced pluripotent cell (iPSC) lines to assess the cardiotoxicity and neurotoxicity of the first line of anti-COVID-19 drugs. We also infected iPSC-derived cells to understand the viral infection of cardiomyocytes and neurons. We found that iPSC-derived cardiomyocytes express the ACE2 receptor which correlated with a higher infection of the SARS-CoV-2 virus (r = 0.86). However, we were unable to detect ACE2 expression in neurons which correlated with a low infection rate. We then assessed the toxicity of anti-COVID-19 drugs and identified two cardiotoxic compounds (remdesivir and arbidol) and four neurotoxic compounds (arbidol, remdesivir, hydroxychloroquine, and chloroquine). These data show that this platform can quickly and easily be employed to further our understanding of cell-specific infection and identify drug toxicity of potential treatment options helping clinicians better decide on treatment options.

## 1. Introduction

In December 2019, the novel coronavirus disease 2019 (COVID-19), caused by severe acute respiratory syndrome coronavirus 2 (SARS-CoV-2), spread from Wuhan, China to the rest of the world within months, affecting almost every country around the world. According to the World Health Organization, as of 1 March 2022, there have been ~435 million confirmed cases and ~6 million related deaths. The COVID-19 pandemic has also caused a devastating crash in economies across all sectors [1].

SARS-CoV-2 is a novel single-stranded enveloped RNA virus and is the seventh known coronavirus to infect humans [2]. The main route of entry of SARS-CoV-2 is through the respiratory tract, manifesting in clinical symptoms such as fever and dry cough [3,4] that are experienced by 88% and 67% of patients, respectively (World Health Organization). However, many patients first report anosmia (loss of smell) or ageusia (loss of taste) [5], which are functions of the olfactory bulb in the brain. Many studies have shown that SARS-CoV-2 establishes itself through the angiotensin-converting enzyme 2 (ACE2) receptor [6], which is mainly expressed in the heart, blood vessels, intestines, kidneys, and pulmonary alveolar (type II) cells [7,8,9]. SARS-CoV-2 infection is initiated by the binding of the viral surface spike protein to the ACE2 receptor following activation of the spike protein by the transmembrane protease serine 2 (TMPRSS2) [6]. 

Although SARS-CoV-2 initially affects the lungs and induces SARS, more evidence has shown that it also affects multiple organs, such as the heart, brain, kidneys, liver, and eyes. As the spread of SARS-CoV-2 continues, there is an emerging trend revealing that patients with underlying cardiovascular disease are disproportionately affected [10]. Similar to the SARS and MERS pandemics, cardiovascular disease is a common comorbidity in COVID-19 patients. Clinical data demonstrate that SARS-CoV-2 infection causes cardiac complications, including increased blood cardiac troponin I levels, an indication of cardiomyocyte death, arrhythmias, and heart failure [11]. 

In addition to its effect on the respiratory and cardiovascular systems, COVID-19 patients exhibit neurological symptoms and potential threat to the nervous system. Similar to other human coronaviruses such as SARS or MERS, COVID-19 has been shown to cause headache, epilepsy, disturbed consciousness, or even cerebral hemorrhages in approximately 36% of COVID-19 patients [12]. This demonstrates that, while COVID-19 predominantly infects the respiratory tract and cardiovascular system, its neuro-invasive characteristics may result in far more detrimental effects on human health, causing pathological effects similar to early tauopathies and neuronal cell death. It has been proposed that the infection of high ACE2-expressing non-neuronal olfactory endothelium cells is a probable starting point for disrupting neuronal functions, which explains the early stage loss of smell [13,14]. Being passed down from non-neuronal olfactory endothelium to olfactory receptor neurons, the virus can be transported along olfactory axons and consequently across the blood-brain barrier into the brain [15]. While this proposed mechanism is referenced in many studies, the neuro-invasive potential of COVID-19 requires further investigation.

Aside from the biological stress imposed by the virus, many of the current treatment options have been known to cause cardiotoxicity [16]. To combat COVID-19 quickly, there have been many attempts to repurpose current FDA-approved drugs or revive old drugs with anti-viral properties [17]. However, this raises a major safety issue as many of these drugs have resulted in adverse drug reactions, or worse, death [18]. These include drugs such as ACE inhibitors, drugs that target the endo-lysosomal pathway such as chloroquine and hydroxychloroquine [19], and antibiotics such as azithromycin. Furthermore, drugs such as remdesivir and chloroquine, which are used to treat malaria, have been widely used for their anti-viral effects [17]. Some of these drugs have known or expected toxicity. As such, the FDA has warned against using hydroxychloroquine and chloroquine outside of a hospital setting or clinical trial due to the risk of heart complications. Therefore, we aim to establish a drug toxicity screening platform using 13 human iPSC lines to investigate the toxicity of the current and prospective COVID-19 treatment options, in particular, those with promising outcomes, to determine their potential cardio- and neurotoxicity in humans.

## 2. Results

To study the toxicity of the repurposed drugs and provide the most relevant platform for drug screening we employed human cells. In a previous study, we generated an induced pluripotent cell (iPSC)-based population drug screening platform using human iPSCs. We generated 13 cell lines that were representative of Taiwans population, based on the high-frequency human leukocyte antigen (HLA) alleles in Taiwan [20]. Using these cell lines, we generated and characterized cardiomyocytes (hiPSC-CMs) and neurons (hiPSC-NEURs). As part of our quality control, we only used cultures that were more than 90% pure cardiomyocytes or neurons assessed by flow cytometry. The cardiomyocytes in our cultures were mainly (~85%) ventricular with approximately ~15% atrial, similar to other studies with a similar protocol [21]. The neurons are mainly (~80%) glutamatergic with a small population (~20% GABAergic) of neurons. The cardiomyocytes and neurons used here have been fully characterized in a previous publication [20]. 

### 2.1. SARS-CoV-2 Virus Infection of iPSC-Derived Cardiomyocytes and Neurons

**Figure 1:**
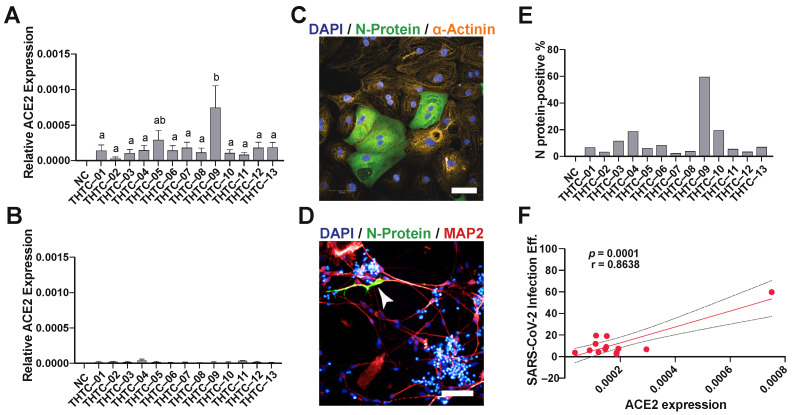
SARS-CoV-2 infects iPSC-cardiomyocytes but not neurons: (**A**,**B**) Quantification of ACE2-mRNA expression by RT-PCR in (**A**) hiPSC-derived cardiomyocytes and (**B**) neurons, n = 3; (**C**,**D**) a representative confocal image of cardiomyocytes (**C**) and neurons (**D**) infected with SARS-CoV-2. Cardiomyocyte scale bar: 50 µm, Neuron scale bar: 20 µm; (**E**) high-content quantification of SARS-CoV-2 infection efficiency in cardiomyocytes; (**F**) correlation between ACE2 expression and infection efficiency in cardiomyocytes. (**E**) Quantification of ACE2 expression by RT-PCR in hiPSC-derived neurons (*n* = 3). (**F**) High-content immunocytochemical analysis of infected SARS-CoV-2 infected neurons.

Due to the development of the COVID-19 pandemic, this platform was then employed to assess the cardiotoxicity and neurotoxicity of anti-SARS-CoV-2 infection drugs and the infection capability of the hiPSC-derived cells. First, we assessed the expression levels of the SARS-CoV-2 receptor, ACE2, in both hiPSC-CMs and hiPSC-NEURs. The RT-PCR results revealed that ACE2 was expressed in all hiPSC-CM lines (Figure 1A); however, in contrast, hiPSC-NEURs had very low expression (Figure 1B). The ACE2 expression in the hiPSC-CM line THTC-09 was significantly higher than other hiPSC-CM lines, except THTC-05. Then, we tested if SARS-CoV-2 could infect hiPSC-CMs and hiPSC-NEURs. The results showed that SARS-CoV-2 infected all hiPSC-CM lines (Figure 1C,E). We were unable to quantify infection efficiency in neurons due to the low infection rate of hiPSC-NEURs. However, we were able to identify a few infected MAP2-positive neurons in our culture (Figure 1D). We then correlated the ACE2 receptor expression to SARS-CoV-2 infection efficiency in hiPSC-CMs, which showed a significant positive correlation with SARS-CoV-2 infection with a correlation coefficient value (r) of 0.8638 (*p* = 0.0001, Figure 1F). 

### 2.2. Drug Screening of Anti-COVID-19 Compounds

**Figure 2:**
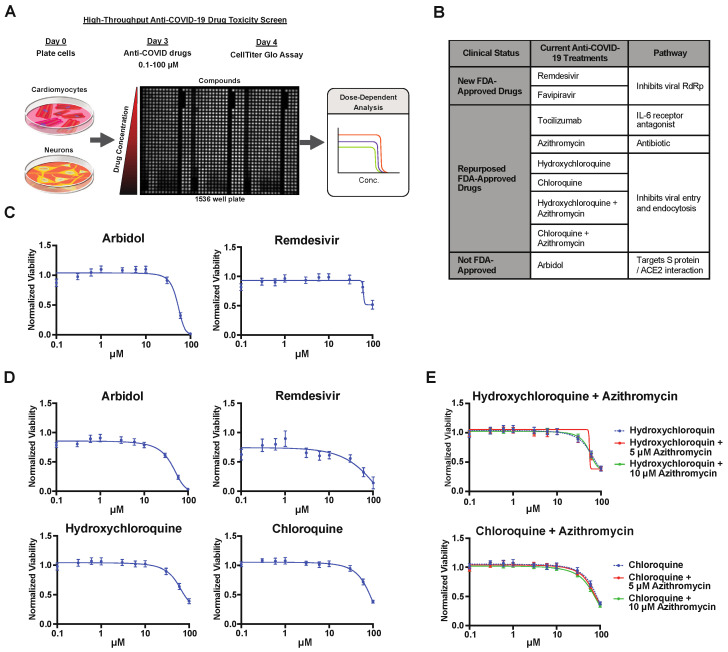
Identification of cardio- and neurotoxic compounds used for the treatment of SARS-CoV-2 infection: (**A**) Workflow of cardio- and neurotoxicity screen using potential anti-COVID-19 compounds with an example image of a 1536-well plate following CellTiter-Glo assay; (**B**) table of selected anti-COVID-19 compounds; (**C**–**E**) comparative evaluation of cell viability by CellTiter-Glo assay following 24-h exposure to COVID-19 treatments in (**C**) hiPSC-CMs and (**D**) hiPSC-NEURs (n = 3). (**E**) Comparative evaluation of cell viability by CellTiter-Glo assay following 24-h exposure to COVID-19 treatments in neurons. All data represent mean ± SEM from the 13 cell lines.

We then applied our drug toxicity platform to test the toxicity of anti-COVID-19 drugs (Figure 2). The schematic diagram shows the workflow of our high-throughput anti-COVID-19 drug toxicity screen (Figure 2A). Our test compounds included some repurposed drugs such as chloroquine, a new drug, remdesivir in clinical trials to improve the clinical outcomes in patients hospitalized with COVID-19, and tocilizumab. A list of selected anti-COVID-19 drugs is shown in Figure 2B. The Z’ value was calculated for each plate with a minimum value Z’ > 0.5 between the positive control (staurosporine) and the negative control (DMSO) as the minimum for the plate to be valid (Figure S1A). Among these nine drug treatments, cardiotoxicity was observed in remdesivir (IC50 = 51 µM) and arbidol (IC50 = 61 µM) treated cells in most of our population-representative lines (Figure 2C and Figure S1B). Similarly, neurotoxicity was observed in remdesivir (IC50 = 41 µM), arbidol (IC50 = 42 µM), hydroxychloroquine (IC50 = 52 µM), and chloroquine (IC50 = 71 µM) treated hiPSC-NEURs (Figure 2D and Figure S1C). We also tested the combined treatment of hydroxychloroquine or chloroquine with azithromycin; however, the addition of either 5 µM or 10 µM of azithromycin did not induce further neurotoxicity when combined with hydroxychloroquine or chloroquine (Figure 2E).

## 3. Discussion

Throughout the COVID-19 pandemic, there have been conflicting results relating to the side effects of anti-COVID-19 drugs. Here, we employed a population-based platform using 13 iPSC lines and derived cardiomyocytes and neurons to perform a population-based drug screen. Many of the previous studies investigating these drugs used non-human cells or a single cell line which would not account for individual differences in drug response [22,23,24,25]. As drug response can be population specific, it is important to understand the drug toxicity levels in different populations. This is commonly seen with diazepam, warfarin, and statins, where the Asian populations are generally prescribed lower doses due to their ability to metabolize the drug [26,27,28,29]. 

It has been reported that COVID-19 patients receiving arbidol treatment of 200 mg three times per day, which is the recommended dosing regime, had an increased risk of in-hospital mortality [30,31]. Another study investigating arbidol found that the plasma concentrations following a single dose of 800 mg was 4.1 µM, which was lower than the EC50 observed in our study. However, arbidol had prolonged plasma concentrations, suggesting that multiple doses or prolonged treatments may increase the plasma concentration [32]. In our study, we observed both cardiotoxicity and neurotoxicity to abidol which may explain why some patients have adverse reactions to these drugs. Another anti-viral drug, remdesivir, was one of the first most promising drugs during the COVID-19 pandemic and continues to be used for newly hospitalized COVID-19 patients. However, many studies report adverse effects of remdesivir on the cardiovascular system such as hypotension, atrial fibrillation rhythm, and sinus bradycardia [33]. Similarly, remdesivir induced both cardio- and neurotoxicity in our hiPSC-derived cells with an average IC50 value of 51 µM and 41 µM for cardiotoxicity and neurotoxicity, respectively. This concentration is similar to the plasma concentration, between 16.7 µM and 1.7 mM, following the clinical dosing regimen, suggesting that the concentration used in our assays was similar to that found clinically [34]. There was also individual variability in the neurotoxicity of remdesivir between individuals as low as 3 µM. This suggests that some patients may be more susceptible to neurological complications when administered remdesivir. Interestingly, recent studies have suggested that remdesivir-treated SARS-CoV-2-infected hiPSC-CMs have more damage as compared with uninfected remdesivir-treated cells [35,36]. However, studies investigating these drugs only employed a single iPSC line [22,23,24,25] and should have used more cell lines to validate a drug response. Our results showed that remdesivir induced toxicity in most, but not all, cell lines, highlighting the inter-individual differences and the potential for inter-population differences. Furthermore, a study investigating the potential of remdesivir to prevent SARS-CoV-2 infection used Vero (African monkey epithelial) and Huh7.5 (human epithelial-like) cells did not observe cell toxicity to remdesivir [25]. This highlights the cell-specific and species-specific toxicity of remdesivir. The authors did also observe toxicity to hydroxychloroquine in both of their cell lines, however, we only observed toxicity in neurons, not cardiomyocytes. This further shows the importance of investigating toxicity in multiple cell types and using human cells. Although remdesivir has shown therapeutic potential in preventing hospitalization in SARS-CoV-2 patients, the question remains whether the toxicity side effect outweighs the therapeutic effect of the drug. Another treatment regime was to combine treatments of hydroxychloroquine or chloroquine with azithromycin. Some, but not all, COVID-19 patients who received the combined treatment of hydroxychloroquine and azithromycin were reported to have prolongation of the QT interval and arrhythmia [37]. In these drug treatments, we did not see any increased toxicity due to combined treatments, however, we did not investigate the potential arrhythmia effects of these drugs. Furthermore, the toxicity of these drugs may be observed at lower doses at extended time points or after multiple doses to detect any latent toxicity. This will be the focus of future work.

Although SARS-CoV-2 is known to cause neurological complications such as the loss of olfactory function and inflammation of brain tissues [38], similar to other studies [23], we found that SARS-CoV-2 did not significantly infect our hiPSC-NEURs. This suggests that the neurological complications may be due to other effects of COVID-19. It has been proposed that the cytokine storm associated with viral infection may cause neurological damage [39] which would not occur in our in vitro conditions. This cytokine storm is manifested by elevated levels of interleukin-6, ferritin, lactate dehydrogenase, and D-dimer. The second complication is the result of direct SARS-CoV-2 infection of the cells causing cell damage and death. Especially in the case of endothelial cells, which are extremely vulnerable to SARS-CoV-2 infection, where the entry of the virus can lead to widespread infections of major organs across the human body. In our study, we only investigated viral infection and drug toxicity in neurons. Previous studies have shown that SARS-CoV-2 can cause damage to the choroid plexus causing damage to the brain barrier [22]. This would be important to take into account as more drugs would be able to enter the brain and potentially cause more damage or toxicity. It is also important to note that these drugs may alter the function of cardiomyocytes or neurons which would not be detected in these assays. It would be important to know if these drugs cause arrhythmia or alter neuron function, but not cause toxicity. 

**Figure 3:**
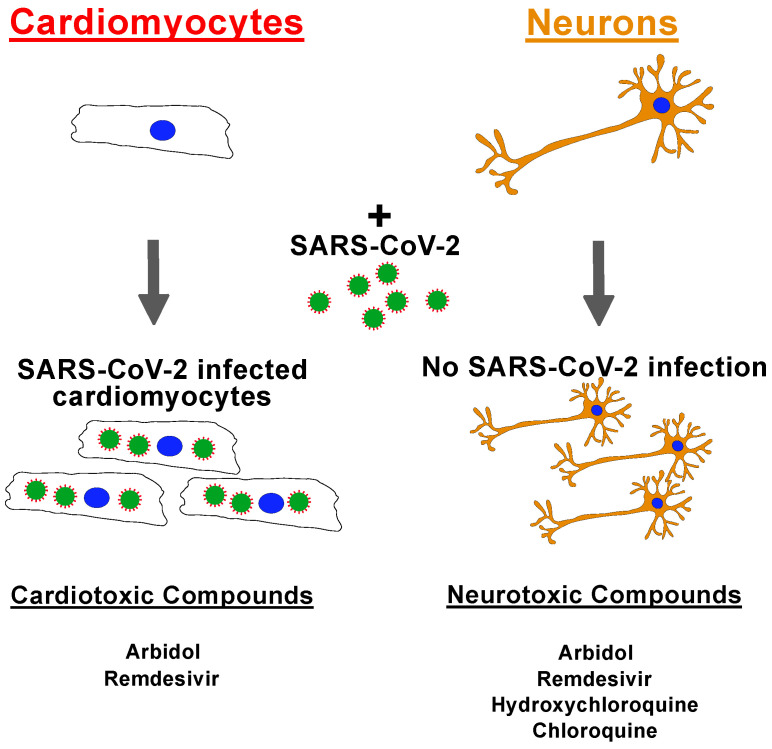
Overview of results showing that cardiomyocytes can be infected with SARS-CoV-2, but not neurons, and a list of cardiotoxic and neurotoxic anti-COVID-19 compounds.

Our data also show that ACE2 expression is cell-type dependent within the same hiPSC line. We showed that cardiomyocytes had ten times higher ACE2 receptor expression than neurons and that one cell line had significantly higher ACE2 expression in cardiomyocytes. ACE2 expression is different between individuals with factors such as race, age, and disease status playing a role in the expression levels [40]; therefore, the individual differences observed are likely due to individual differences rather than differences caused by the differentiation protocol. Previous studies have also shown that, apart from ACE2 receptors, certain HLA haplotypes have been reported to have higher infection rates. For example, HLA-B*46:01 may be particularly vulnerable to COVID-19 [41]; however, we found that hiPSC-CMs with this allele (THTC-02, THTC-10, and THTC-13) were not significantly higher in SARS-CoV-2 infections. Furthermore, a study in China showed that HLA-C*07:29 and HLA-B*15:27 were more frequently present in COVID-19 patients [42]; however, these alleles are rare in the Chinese population. In an Italian study, the HLA-A*01:01 allele was positively correlated with COVID-19 infection, while HLA-A*02:01 was negatively correlated [43]. Our results revealed that the hiPSC-CM line THTC-09 (HLA-A*33:03, HLA-B*58:01, HLA-C*03:02, and HLA-DRB1*03:01) was more susceptible to SAR-CoV-2 infection which correlated with the high level of ACE2 receptor expression. This may suggest that individuals with this haplotype may be more susceptible to cardiovascular complications due to viral infection. These studies are all correlative studies and have yet to show any direct causative evidence. This study shows the cardio- and neurotoxicity of the common anti-COVID-19 drugs using a robust platform employing 13 iPSC lines derived into cardiomyocytes or neurons and their infection with SARS-CoV-2 (Figure 3). We also show there are inter-individual differences in drug response to remdesivir in specific cell types. Importantly, this platform allows for toxicity testing in different cell types, and in the case of any future pandemics or outbreaks, these cells can be rapidly deployed to investigate specific toxicities to potential treatment options. 

### Limitations of Study

All handling of the virus requires a P3 level facility, however, due to the location of the high-throughput and liquid handling equipment outside the P3 facility, we were unable to investigate the effect of drug toxicity on cells infected with SARS-CoV-2. In addition, as the brain contains a diverse group of cells, we were unable to screen all cell types such as endothelial cells, microglial cells, and astrocytes. Since neurons are the main cell type, we focused our efforts on neurons. 

## 4. Materials and Methods

### 4.1. Immunocytochemistry

Immunocytochemistry was performed as previously described [44]. Briefly, cells were fixed in 4% paraformaldehyde, and then permeabilized using 0.1% Triton-X100 when required. Specific primary antibodies were incubated overnight. Subsequently, appropriate Alexa 594-, Alexa 488-, and Alexa 647-conjugated secondary antibodies (Molecular Probes) were incubated for 1 h at room temperature. Imaging was captured using a Zeiss LSM 700 confocal microscope (Carl Zeiss) and processed through the Zen imaging software. 

### 4.2. Human iPSCs

HLA homozygous hiPSCs were generated and provided by the Taiwan Human Disease iPS Cells Service Consortium (accessed on 18 June 2022 http://ipsc.ibms.sinica.edu.tw/index_e.html). They were cultured according to a previously published protocol [20].

### 4.3. Cardiac Differentiation

HiPSCs were differentiated as previously described [31]. In brief, hiPSCs were seeded on a Matrigel-coated plate. Once cells reached ~80% confluency, the cells were then treated with 6 to 10 µM CHIR99021 (Selleckchem; Berlin, Germany) to active Wnt signaling to induce mesoderm differentiation. Then, the medium was refreshed to RPMI/B27 insulin-free medium (Thermo Fisher; Waltham, MA, USA) on Day 2. On Day 3, the cells were then treated with 5 µM IWR-1 (Sigma; St. Louis, MO, USA) for a further 48 h. After two days, the medium was changed to remove IWR-1. Then, the cells were cultured in RPMI/B27 medium until the cells started to beat, and then they cultured in glucose-free RPMI/B27 for purification. 

### 4.4. Neuronal Differentiation

Neuronal cells were differentiated according to a previous protocol [20]. To obtain neuronal stem cells (hiPSC-NSCs), hiPSCs were plated in StemFlex medium on Matrigel-coated 6-well plates. The following day, the medium was replaced with neuronal induction medium (neurobasal medium (Thermo Fisher; Waltham, MA, USA) and 2% neuronal induction supplement (Thermo Fisher; Waltham, MA, USA)). The cells were cultured for 7 days, and then cultured in neuronal expansion medium (50% neurobasal medium, 50% DMEM/F:12 (Thermo Fisher), 2% neuronal induction supplement) for a further 7 days. To generate neurons, hiPSC-NSCs were plated onto poly-L-ornithine (0.1 mg/mL) and laminin (10 µg/mL) coated 6-well plates in StemPro medium (Knockout DMEM/F:12 (Thermo Fisher), 2% StemPro neuronal supplement (Thermo Fisher), L-glutamine, 10 µg/mL bFGF, 10 µg/mL EGF) for 2 days. The medium was changed to neurobasal medium (neurobasal medium, 2% B27 (Gibco; Waltham, MA, USA), L-glutamine) for a further 21 days with half medium change every 3 days. 

### 4.5. ACE2 Expression

For real-time polymerase chain reaction, RNA was extracted from all 13 lines of representative hiPSC-CMs or hiPSC-NEURs. Five-hundred ng RNA was reverse transcribed by Superscript IV reverse transcriptase (Thermo Fisher). Then, cDNA was used for the SYBR green PCR to detect expression of ACE2, and the housekeeping β-actin gene was used for normalization. The primers are listed in Table S1. 

### 4.6. SARS-CoV-2 Infection

All experiments using SARS-CoV-2 (TCDC#4, sequence available on the GISAID website) were performed in the Biosafety Level 3 (BSL-3) facility at Institute Biomedical Sciences Academia Sinica. Frst, 45,000 hiPSC-NEURs were plated in 96-well plates and infected with SARS-CoV-2 at a MOI = 0.1 for 48 h at 37 °C. Then, the cells were fixed with 4% paraformaldehyde and permeabilized with 0.1% Triton X-100. A polyclonal antibody against SARS-CoV-2 nucleocapsid protein 1:2000 (Sino Biological, 40143-R001-50) was used to detect virus expression, the infection efficiency was carried out through an Opera Phenix™ High-Content Screening System (PerkinElmer) using the whole-well imaging method to quantify infection rate. 

### 4.7. Toxicity Screening of Anti-COVID-19 Drugs

The anti-COVID-19 compounds used in the study included remdesivir (Cas No. 1809249-37-3), arbidol hydrochloride (Cas No. 131707-23-8), favipiravir (Cas No. 259793-96-9), tocilizumab (Cas No. 375823-41-9), azithromycin (Cas No. 83905-01-5), hydroxychloroquine (Cas No. 118-42-3), and chloroquine (Cas No. 54-05-7; all form TargetMol). Ten concentrations from 0.1 µM to 100 µM of various drugs were used to generate a dose-dependent curve to investigate drug-induced toxicity in hiPSC-CMs and hiPSC-NEURs. All representative cell lines were treated with various dosages of anti-COVID-19 drugs. Cell toxicity was determined through CellTiter-Glo^®^ Luminescent Cell Viability Assay (Promega) 24 h after drug treatment.

### 4.8. Statistical Analysis

The data are presented as mean ± standard error of the mean. Multiple comparisons were analyzed by ANOVA followed by Bonferroni post hoc analysis, while two groups were analyzed by unpaired two-tailed Student’s *t*-test with the Graphpad Prism software (version 9.1.1; San Diego, CA, USA). *p-*value <0.05 was considered to be statistically significant.

## Data Availability

All materials generated in this study are available upon request from the lead contact and upon signature of the corresponding Material Transfer Agreement, if necessary.

## Supplementary Materials

The following supporting information can be downloaded at: https://www.mdpi.com/article/10.3390/ph15060765/s1, Figure S1: Anti-COVID-19 drug toxicity. (A) Example of luminescence values from a 1536 well plate treated with anti-COVID compounds, positive control (staurosporine; red), and negative control (1% DMSO; green) with Z’ calculation. Comparative evaluation of cell viability by CellTiter-Glo assay of 13 HLA-homozygous hiPSC-CM (B) and hiPSC-NEUR (C) following 24-h exposure to anti-COVID-19 drugs. n = 3 for each drug concentration. Data represents mean ± SEM.; Table S1: Primers used in PCR experiments.

## References

[1] Mehraeen E., Behnezhad F., Salehi M.A., Noori T., Harandi H., SeyedAlinaghi S. (2021). Olfactory and gustatory dysfunctions due to the coronavirus disease (COVID-19): A review of current evidence. Eur. Arch. Otorhinolaryngol..

[2] Pal M., Berhanu G., Desalegn C., Kandi V. (2020). Severe Acute Respiratory Syndrome Coronavirus-2 (SARS-CoV-2): An Update. Cureus.

[3] Liu J., Liao X., Qian S., Yuan J., Wang F., Liu Y., Wang Z., Wang F.S., Liu L., Zhang Z. (2020). Community Transmission of Severe Acute Respiratory Syndrome Coronavirus 2, Shenzhen, China, 2020. Emerg. Infect. Dis..

[4] Li J., Gong X., Wang Z., Chen R., Li T., Zeng D., Li M. (2020). Clinical features of familial clustering in patients infected with 2019 novel coronavirus in Wuhan, China. Virus Res..

[5] Hoffmann M., Kleine-Weber H., Schroeder S., Kruger N., Herrler T., Erichsen S., Schiergens T.S., Herrler G., Wu N.H., Nitsche A. (2020). SARS-CoV-2 Cell Entry Depends on ACE2 and TMPRSS2 and Is Blocked by a Clinically Proven Protease Inhibitor. Cell.

[6] Nicin L., Abplanalp W.T., Mellentin H., Kattih B., Tombor L., John D., Schmitto J.D., Heineke J., Emrich F., Arsalan M. (2020). Cell type-specific expression of the putative SARS-CoV-2 receptor ACE2 in human hearts. Eur. Heart J..

[7] Rice G.I., Thomas D.A., Grant P.J., Turner A.J., Hooper N.M. (2004). Evaluation of angiotensin-converting enzyme (ACE), its homologue ACE2 and neprilysin in angiotensin peptide metabolism. Biochem. J..

[8] Xiao L., Haack K.K., Zucker I.H. (2013). Angiotensin II regulates ACE and ACE2 in neurons through p38 mitogen-activated protein kinase and extracellular signal-regulated kinase 1/2 signaling. Am. J. Physiol. Cell Physiol..

[9] Guo T., Fan Y., Chen M., Wu X., Zhang L., He T., Wang H., Wan J., Wang X., Lu Z. (2020). Cardiovascular Implications of Fatal Outcomes of Patients With Coronavirus Disease 2019 (COVID-19). JAMA Cardiol..

[10] Shi S., Qin M., Shen B., Cai Y., Liu T., Yang F., Gong W., Liu X., Liang J., Zhao Q. (2020). Association of Cardiac Injury With Mortality in Hospitalized Patients With COVID-19 in Wuhan, China. JAMA Cardiol..

[11] Wu Y., Xu X., Chen Z., Duan J., Hashimoto K., Yang L., Liu C., Yang C. (2020). Nervous system involvement after infection with COVID-19 and other coronaviruses. Brain Behav. Immun..

[12] Ramani S., Berard J.A., Walker L.A.S. (2021). The relationship between neurofilament light chain and cognition in neurological disorders: A scoping review. J. Neurol. Sci..

[13] Varga Z., Flammer A.J., Steiger P., Haberecker M., Andermatt R., Zinkernagel A.S., Mehra M.R., Schuepbach R.A., Ruschitzka F., Moch H. (2020). Endothelial cell infection and endotheliitis in COVID-19. Lancet.

[14] Brann D.H., Tsukahara T., Weinreb C., Lipovsek M., Van den Berge K., Gong B., Chance R., Macaulay I.C., Chou H.J., Fletcher R.B. (2020). Non-neuronal expression of SARS-CoV-2 entry genes in the olfactory system suggests mechanisms underlying COVID-19-associated anosmia. Sci. Adv..

[15] Butowt R., von Bartheld C.S. (2021). Anosmia in COVID-19: Underlying Mechanisms and Assessment of an Olfactory Route to Brain Infection. Neuroscientist.

[16] Yanagida S., Satsuka A., Hayashi S., Ono A., Kanda Y. (2021). Comprehensive Cardiotoxicity Assessment of COVID-19 Treatments Using Human-Induced Pluripotent Stem Cell-Derived Cardiomyocytes. Toxicol. Sci..

[17] Wang M., Cao R., Zhang L., Yang X., Liu J., Xu M., Shi Z., Hu Z., Zhong W., Xiao G. (2020). Remdesivir and chloroquine effectively inhibit the recently emerged novel coronavirus (2019-nCoV) in vitro. Cell Res..

[18] Wong A.Y., MacKenna B., Morton C.E., Schultze A., Walker A.J., Bhaskaran K., Brown J.P., Rentsch C.T., Williamson E., Drysdale H. (2021). Use of non-steroidal anti-inflammatory drugs and risk of death from COVID-19: An OpenSAFELY cohort analysis based on two cohorts. Ann. Rheum. Dis..

[19] Kalra R.S., Tomar D., Meena A.S., Kandimalla R. (2020). SARS-CoV-2, ACE2, and Hydroxychloroquine: Cardiovascular Complications, Therapeutics, and Clinical Readouts in the Current Settings. Pathogens.

[20] Huang C.Y., Nicholson M.W., Wang J.Y., Ting C.Y., Tsai M.H., Cheng Y.C., Liu C.L., Chan D.Z.H., Lee Y.C., Hsu C.C. (2022). Population-based high-throughput toxicity screen of human iPSC-derived cardiomyocytes and neurons. Cell Rep..

[21] Hwang H.S., Kryshtal D.O., Feaster T.K., Sanchez-Freire V., Zhang J., Kamp T.J., Hong C.C., Wu J.C., Knollmann B.C. (2015). Comparable calcium handling of human iPSC-derived cardiomyocytes generated by multiple laboratories. J. Mol. Cell Cardiol..

[22] Pellegrini L., Albecka A., Mallery D.L., Kellner M.J., Paul D., Carter A.P., James L.C., Lancaster M.A. (2020). SARS-CoV-2 Infects the Brain Choroid Plexus and Disrupts the Blood-CSF Barrier in Human Brain Organoids. Cell Stem Cell.

[23] Simoneau C.R., Ott M. (2020). Modeling Multi-organ Infection by SARS-CoV-2 Using Stem Cell Technology. Cell Stem Cell.

[24] Yang L., Han Y., Nilsson-Payant B.E., Gupta V., Wang P., Duan X., Tang X., Zhu J., Zhao Z., Jaffre F. (2020). A Human Pluripotent Stem Cell-based Platform to Study SARS-CoV-2 Tropism and Model Virus Infection in Human Cells and Organoids. Cell Stem Cell.

[25] Dittmar M., Lee J.S., Whig K., Segrist E., Li M., Kamalia B., Castellana L., Ayyanathan K., Cardenas-Diaz F.L., Morrisey E.E. (2021). Drug repurposing screens reveal cell-type-specific entry pathways and FDA-approved drugs active against SARS-Cov-2. Cell Rep..

[26] Kim K., Johnson J.A., Derendorf H. (2004). Differences in drug pharmacokinetics between East Asians and Caucasians and the role of genetic polymorphisms. J. Clin. Pharmacol..

[27] Lin K.M., Lau J.K., Smith R., Phillips P., Antal E., Poland R.E. (1988). Comparison of alprazolam plasma levels in normal Asian and Caucasian male volunteers. Psychopharmacology.

[28] Dang M.-T.N., Hambleton J., Kayser S.R. (2005). The Influence of Ethnicity on Warfarin Dosage Requirement. Ann. Pharmacother..

[29] Liao J.K. (2007). Safety and efficacy of statins in Asians. Am. J. Cardiol..

[30] Mercuro N.J., Yen C.F., Shim D.J., Maher T.R., McCoy C.M., Zimetbaum P.J., Gold H.S. (2020). Risk of QT Interval Prolongation Associated With Use of Hydroxychloroquine With or Without Concomitant Azithromycin Among Hospitalized Patients Testing Positive for Coronavirus Disease 2019 (COVID-19). JAMA Cardiol..

[31] Zhou X., Hou H., Yang L., Ding G., Wei T., Li C., Heng Y., Liu R., Ma M., Hu Z. (2021). Arbidol is associated with increased in-hospital mortality among 109 patients with severe COVID-19: A multicenter, retrospective study. J. Glob. Health.

[32] Sun Y., He X., Qiu F., Zhu X., Zhao M., Li-Ling J., Su X., Zhao L. (2013). Pharmacokinetics of single and multiple oral doses of arbidol in healthy Chinese volunteers. Int. J. Clin. Pharmacol. Ther..

[33] Nabati M., Parsaee H. (2022). Potential Cardiotoxic Effects of Remdesivir on Cardiovascular System: A Literature Review. Cardiovasc. Toxicol..

[34] Humeniuk R., Mathias A., Kirby B.J., Lutz J.D., Cao H., Osinusi A., Babusis D., Porter D., Wei X., Ling J. (2021). Pharmacokinetic, Pharmacodynamic, and Drug-Interaction Profile of Remdesivir, a SARS-CoV-2 Replication Inhibitor. Clin. Pharmacokinet..

[35] Perez-Bermejo J.A., Kang S., Rockwood S.J., Simoneau C.R., Joy D.A., Silva A.C., Ramadoss G.N., Flanigan W.R., Fozouni P., Li H. (2021). SARS-CoV-2 infection of human iPSC-derived cardiac cells reflects cytopathic features in hearts of patients with COVID-19. Sci. Transl. Med..

[36] Jacob F., Pather S.R., Huang W.K., Zhang F., Wong S.Z.H., Zhou H., Cubitt B., Fan W., Chen C.Z., Xu M. (2020). Human Pluripotent Stem Cell-Derived Neural Cells and Brain Organoids Reveal SARS-CoV-2 Neurotropism Predominates in Choroid Plexus Epithelium. Cell Stem Cell.

[37] Eftekhar S.P., Kazemi S., Barary M., Javanian M., Ebrahimpour S., Ziaei N. (2021). Effect of Hydroxychloroquine and Azithromycin on QT Interval Prolongation and Other Cardiac Arrhythmias in COVID-19 Confirmed Patients. Cardiovasc. Ther..

[38] Varatharaj A., Thomas N., Ellul M.A., Davies N.W.S., Pollak T.A., Tenorio E.L., Sultan M., Easton A., Breen G., Zandi M. (2020). Neurological and neuropsychiatric complications of COVID-19 in 153 patients: A UK-wide surveillance study. Lancet Psychiatry.

[39] Isacson O. (2020). The Consequences of Coronavirus-Induced Cytokine Storm Are Associated With Neurological Diseases, Which May Be Preventable. Front. Neurol..

[40] Chen J., Jiang Q., Xia X., Liu K., Yu Z., Tao W., Gong W., Han J.J. (2020). Individual variation of the SARS-CoV-2 receptor ACE2 gene expression and regulation. Aging Cell.

[41] Nguyen A., David J.K., Maden S.K., Wood M.A., Weeder B.R., Nellore A., Thompson R.F. (2020). Human Leukocyte Antigen Susceptibility Map for Severe Acute Respiratory Syndrome Coronavirus 2. J. Virol..

[42] Wang W., Zhang W., Zhang J., He J., Zhu F. (2020). Distribution of HLA allele frequencies in 82 Chinese individuals with coronavirus disease-2019 (COVID-19). Hla.

[43] Pisanti S., Deelen J., Gallina A.M., Caputo M., Citro M., Abate M., Sacchi N., Vecchione C., Martinelli R. (2020). Correlation of the two most frequent HLA haplotypes in the Italian population to the differential regional incidence of Covid-19. J. Translational Med..

[44] Huang C.Y., Li L.H., Hsu W.T., Cheng Y.C., Nicholson M.W., Liu C.L., Ting C.Y., Ko H.W., Syu S.H., Wen C.H. (2020). Copy number variant hotspots in Han Taiwanese population induced pluripotent stem cell lines—Lessons from establishing the Taiwan human disease iPSC Consortium Bank. J. Biomed. Sci..

