# Supplementary material for: Cardio- and Neurotoxicity of Selected Anti-COVID-19 Drugs
Source: Pharmaceuticals (Basel). 2022 Jun 20;15(6):765. doi: 10.3390/ph15060765 (PMC9231250; doi:10.3390/ph15060765)
Supplement: Supplementary file 1 [file pharmaceuticals-15-00765-s001.zip › pharmaceuticals-1772505-supplementary.pdf]

Supplementary Materials:

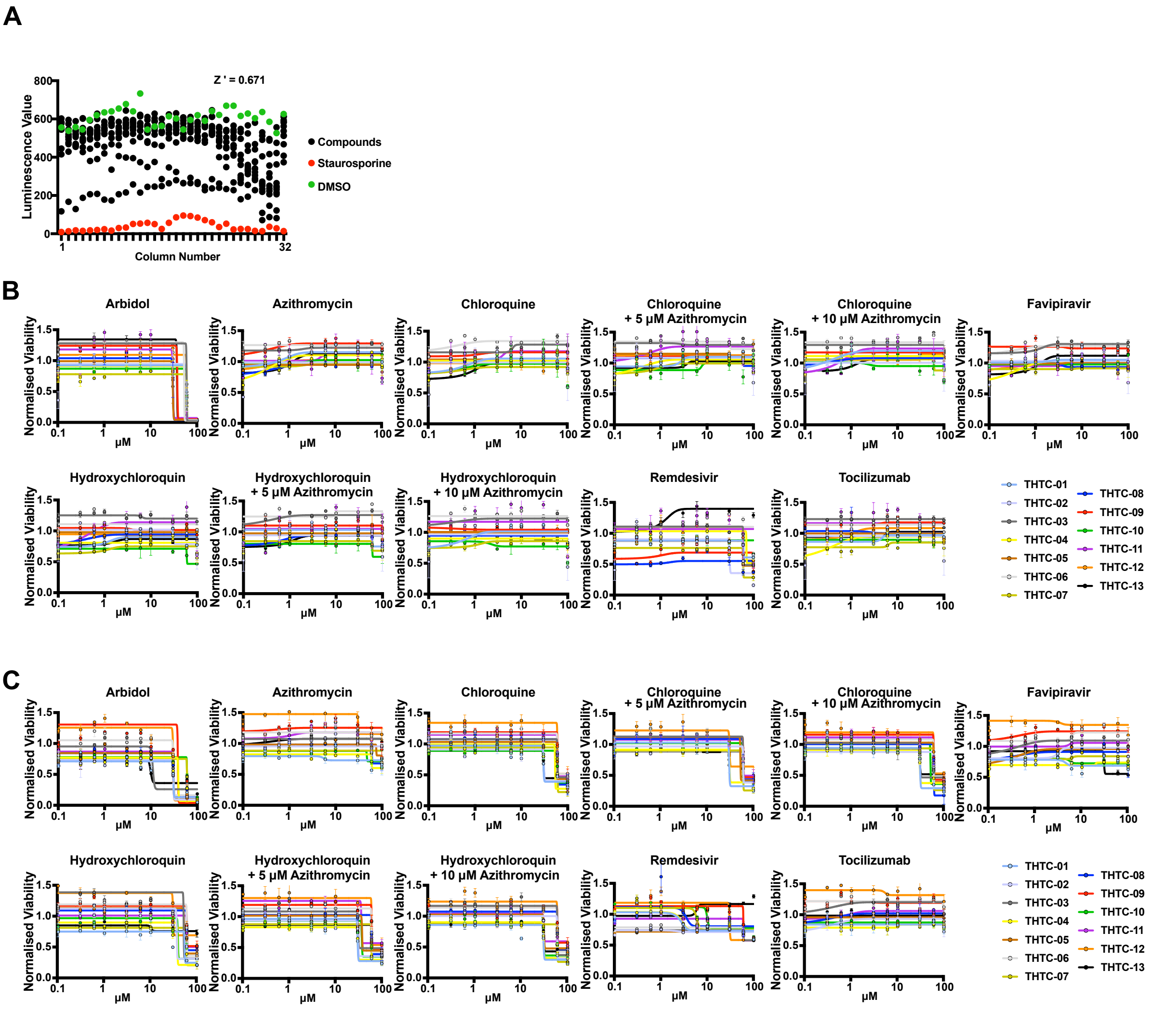

Figure S1. Anti-COVID-19 drug toxicity. (A) Example of luminescence values from a 1536 well plate treated with anti-COVID compounds, positive control (staurosporine; red), and negative control (1% DMSO; green) with Z′ calculation. Comparative evaluation of cell viability by CellTiter-Glo assay of 13 HLA-homozygous hiPSC-CM (B) and hiPSC-NEUR (C) following 24-hour exposure to anti-COVID-19 drugs. n = 3 for each drug concentration. Data represents mean ± SEM.

***Table S1**. Primers used in PCR experiments.*

Target		Sequence
ACE2	Forward	GGGATCAGAGATCGGAAGAAGAAA
	Reverse	AGGAGGTCTGAACATCATCAGTG
Actin	Forward	CCCTGGACTTCGAGCAAGAG
	Reverse	ACTCCATGCCCAGGAAGGAA
